# The {010} form of polar brushite (CaHPO_4_·2H_2_O) deposits as an epi-growing crystal on a non-polar {010} substrate of gypsum (CaSO_4_·2H_2_O)

**DOI:** 10.1107/S1600576725005060

**Published:** 2025-07-16

**Authors:** Dino Aquilano, Stefano Ghignone, Marco Bruno

**Affiliations:** a Dipartimento di Scienze della Terra Università degli Studi di Torino Via Valperga Caluso 35 10125 Torino Italy; b NIS, Centre for Nanostructured Interfaces and Surfaces Università degli Studi di Torino Via G. Quarello 15/a 10135 Torino Italy; Instituto Andaluz de Ciencias de la Tierra, Granada, Spain

**Keywords:** cystal growth, gypsum/brushite pinacoids, gypsum/brushite transformation epitaxy, specific adhesion energy

## Abstract

The mechanisms of crystal growth from solution are more complicated than those from vapor or melt growth. These mechanisms are important in bio­mineralization (bones, teeth, shells) and in the characterization of optically non-linear crystals, perfectly crystallized proteins, nucleic acids and complex viruses. This motivated studies on new basic phenomena, including the crystal interfaces of biological macromolecules and those of low-solubility inorganic salts. Generalization of the Gibbs–Thomson effect has been questioned when describing isotropic (circular) or anisotropic (polygonized) spiral steps spreading onto a crystal face. This work focuses on the brushite crystal (CaHPO_4_·2H_2_O) since it can grow freely or as a deposit on a gypsum (CaSO_4_·2H_2_O) substrate. Brushite looks like a 2D crystal built by a flat {010} form limited by {*h*0*l*} polar and thin faces running as straight steps. Moreover, it is worth investigating the {010} gypsum to {010} brushite transformation since mineralogy and geochemistry play an important role in the crystal growth. Here, we study the freely growing brushite and, subsequently, its {010} form arising from gypsum. The epitaxy {010} gypsum/{010} brushite is also examined because brushite twins are involved. A new way of thinking about the epi-relations is proposed, along with more rigorous constraints; this new approach emphasizes the physical importance of the adhesion energy connected to every epitaxy.

## Abstract 2

Epi-growth of the {010} form of polar brushite (CaHPO_4_·2H_2_O) on the {010} form of non-polar gypsum (CaSO_4_·2H_2_O) is described.

## 1. Introduction

Much research has been carried out on gypsum (CaSO_4_·2H_2_O) due to its abundance and its unique and well understood mineralogy, petrography and geochemistry. The same cannot be said for the less celebrated brushite (CaHPO_4_·2H_2_O). This polar crystal represents an intermediate phase in the natural transformation monetite–brushite–monoclinic hydroxyapatite (HAp), as was shown by Pastero & Aquilano (2016 ▸) for Ca-HAp and by Ma & Liu (2009 ▸), who published the first study where the monoclinic HAp Ca_20_(OH)_4_(PO_4_)_12_ was obtained from brushite at low temperature instead of the more common hexagonal phase.

The meaning of ‘intermediate phase’ is similar to that of ‘transition crystal’, which was originally used to describe the transformation gypsum–brushite–pharmacolite (CaHAsO_4_·2H_2_O) by Heijnen & Hartman (1991 ▸); in that article they compared the structures and the mutual energy aspects of these mineralogical compounds. The role of brushite as a transition crystal in the gypsum–pharmacolite system was re-considered and analyzed in detail by Rodríguez-Blanco *et al.* (2007 ▸).

Today, surface and kinetic quantities, such as specific surface (γ_*hkl*_) and specific adhesion (β_adh_) energies, are no longer evaluated through the distribution of point charges. Instead, changes in crystal habit for a given crystal substance are judged more quantitatively by using its equilibrium (ES) or growth (GS) shapes.

For these reasons and because gypsum is a good substrate for brushite crystallization as a deposit, we need to distinguish both the ES from the GS and the {*hkl*} calculated shapes from the experimental ones. The calculated ES of gypsum is dominated by its {010} pinacoid. In fact, at *T* = 0 K, the gypsum specific surface energy γ_{010}_ reaches 432 erg cm^−2^, whereas the other important forms have higher γ values: γ_{120}_ = 621 erg cm^−2^, γ_{011}_ = 773 erg cm^−2^ and γ_{111}_ = 1027 erg cm^−2^. It turns out that only the {100} pinacoid appears among the calculated (Massaro *et al.*, 2010 ▸; Aquilano *et al.*, 2016 ▸) and drawn {*h*0*l*} forms of the gypsum ES. This result may look surprising, but due to the high γ_{001}_ and 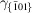 values, neither of these forms in the zone with *A*_2_ // [010] axis is expected to occur in its ES.

Clearly, predicting the gypsum ES alone is not sufficient to understand the complex relations governing the gypsum–brushite transformation; it would be better to relate other growth conditions [*e.g.* temperature (*T*), pressure (*P*) and supersaturation] to crystal habits and determine how such factors affect the growth mechanism and kinetics of crystal faces. This is particularly needed since there are many brushite twinning laws generated at the {010} gypsum/brushite interface, where the more serious problem is how to reconcile the non-polar *A*2/*a* space group of gypsum with the polar one (*Aa*) of brushite.

Atomic force microscopy (AFM) patterns measuring the kinetics of the {010} surfaces of gypsum in aqueous solution showed both a layer-by-layer growth and mono-steps parallel to gypsum [001], [100] and [101] moving with a distinctive anisotropy (Bosbach & Rammensee, 1994 ▸; Bosbach & Hochella, 1996 ▸). Isolated [100] mono-steps move up to 30.0 nm s^−1^, whereas [101] and [001] steps move up to 2.5 nm s^−1^, in an undersaturated aqueous solution (9.8 mmol L^−1^); kinks can also be observed, their formation energy along [001] monolayered steps being 4.1 ± 0.7 kJ mol^−1^ in saturated aqueous solution. These results were the first sign of linear steps running on the {010} gypsum surfaces and, implicitly, that {100}, {001} and {101} pinacoids might be legitimate forms of the gypsum GS.

Van Driessche *et al.* (2010 ▸) were the first to measure the kinetics of {010} gypsum in aqueous solution under controlled *T* and *P*, for both 2D nuclei and spiral growth. Using laser confocal differential interference contrast microscopy and AFM, these authors were able to measure step heights from *h* = ½*d*_010_ to *h* = 16(½*d*_010_), so proving the existence of the {100}, {001} and {101} growth forms.

**Figure 1:**
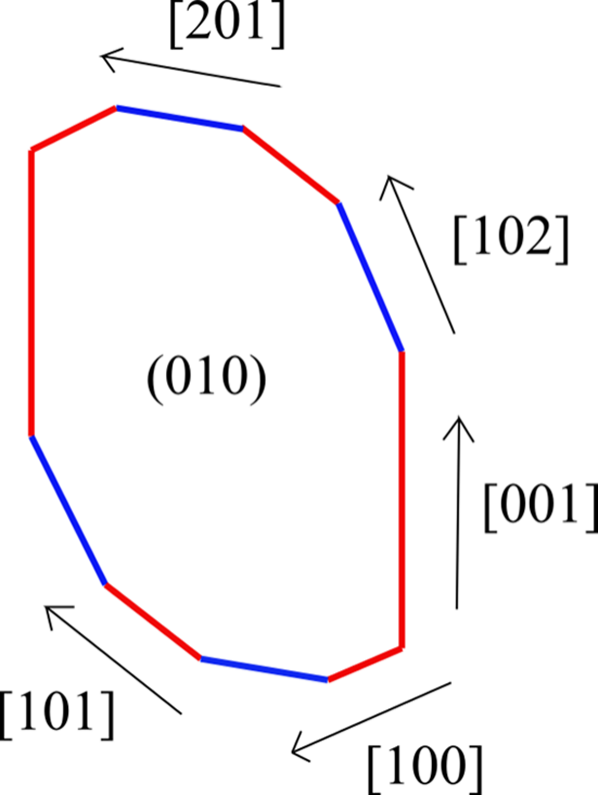
The ES of 2D {010} gypsum, drawn by using ρ_[*uvw*]_ (the main step-free energies, expressed in erg cm^−1^ ± standard deviation): ρ_[001]_ = 8 ± 4, ρ_[102]_ = 16 ± 6, ρ_[101]_ = 25 ± 6, ρ_[201]_ = 50 ± 20, ρ_[100]_ = 23 ± 7. Red lines represent the straight steps, whilst the blue lines stand for kinked ones.

Almost simultaneously, Pinto *et al.* (2010 ▸) studied dissolved gypsum crystals and found epitaxially growing brushite on the gypsum cleaved {010} surfaces. They observed that a layer-by-layer mechanism is always dominant, being controlled by the retreat movements of [001], [101] and [100] steps with a height of ∼7.60 Å, corresponding to *h* = ½*d*_010_. Steps can be isolated but ‘generally delimited etch pits’ that are elongated along [001], with the identity of the [101] and [100] directions frequently lost, mostly developing curved and non-singular tips (Gibbs–Thomson effect). The most advanced AFM research on gypsum {010} has very recently been done in our laboratory, and the entire growth process of spiral hillocks has been observed for the first time with a resolution high enough to measure step spacing (Criado-Reyes *et al.*, 2020 ▸). Criado-Reyes *et al.* (2020 ▸) theoretically studied the {010} gypsum patterns from pure aqueous solution under well controlled kinetic parameters (Fig. 1 ▸).

Knowledge of the morphology of free-growing brushite crystals has been obtained not from the natural world but from laboratory work and has been carefully described over a long period of time (Heijnen & Hartman, 1991 ▸; Le Geros & Le Geros, 1972 ▸; Lundager Madsen & Thorvardarson, 1984 ▸; Lundager Madsen, 2008 ▸; Abbona *et al.*, 1993 ▸; Abbona *et al.*, 1994 ▸; Rinaudo *et al.*, 1994 ▸; Rinaudo *et al.*, 1996 ▸; De Yoreo, 2003 ▸; Pinto *et al.*, 2009 ▸). The morphological importance (MI) of the flat (F) forms {010}, {011}, {011}, {111}, {120} and {122} has been reported by Heijnen & Hartman (1991 ▸). It also seems appropriate to remember a somewhat forgotten article by Abbona *et al.* (1994 ▸), who reconsidered the correct polarity of two synthetic brushite samples. From their studies, a new MI order resulted: {010}; {120} > {120}; {111} > {111}; {111} > {111}; {122} > {122}; and {011} > {011}. The polarization of crystal surfaces explained the different development of the complementary forms {*hkl*} and {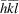}, as was shown by Monier & Kern (1956 ▸). The tabular habit of brushite is due to the flat character of {010}, structurally covered by H_2_O molecules. The other forms are much less important and do not exhibit H_2_O molecules at their surfaces, but mainly Ca^2+^ ions, oxygens of HPO_4_^2−^ or hydrogens of the hydroxyl groups.

This work focuses on the growth of a brushite crystal as a deposit on a gypsum substrate. A theoretical approach is adopted to elucidate this growth mechanism.

The parameters and space groups that we will adopt have been chosen from within the gypsum (Massaro *et al.*, 2010 ▸) and gypsum/brushite (Pinto *et al.*, 2009 ▸) literature. A well defined choice of parameters is vital when one aims to study the relations between two (or more) crystalline substances: the epi-relation between them requires that there be no crystallographic ambiguities, even if the standard deviations on parameters are not needed for our purposes. From X-ray measurements on brushite (in Å), we use in this paper *a*_0_ = 5.812, *b*_0_ = 15.180, *c*_0_ = 6.359; β = 118.52° (space group *Aa*).

**Figure 2:**
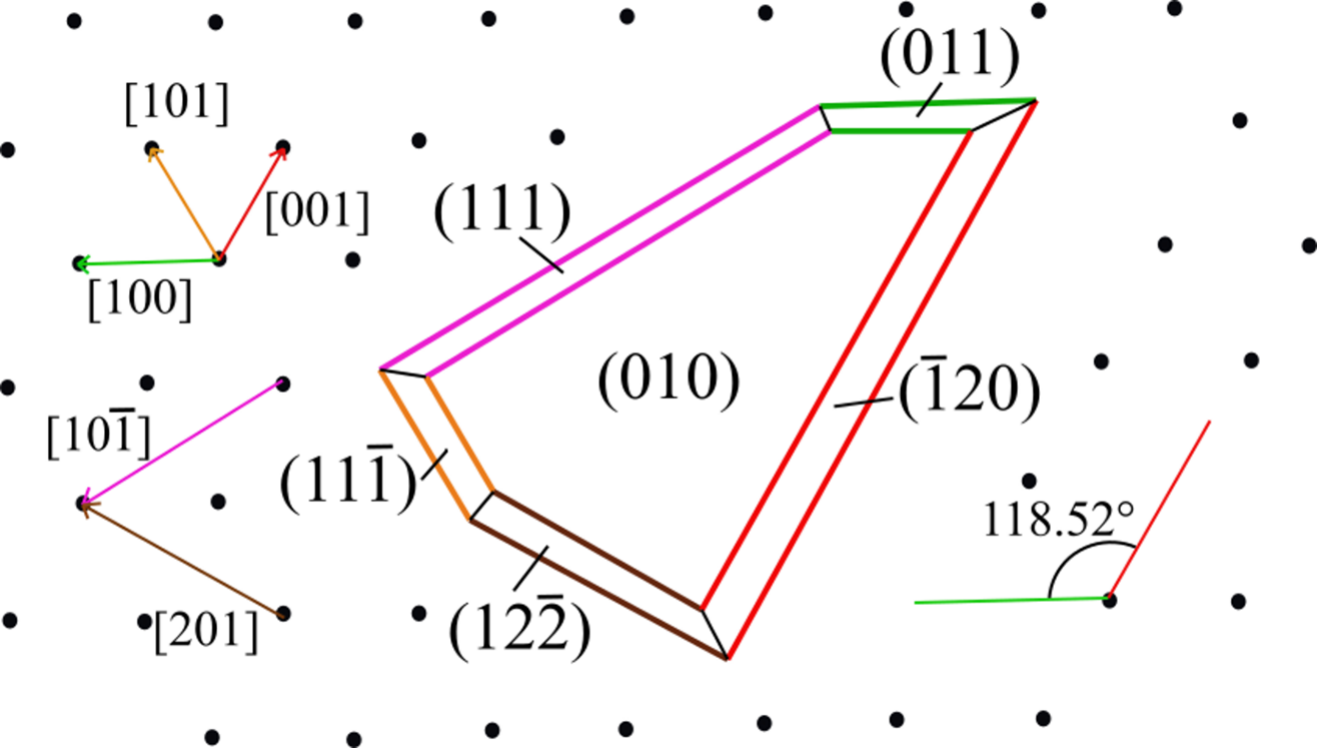
Drawing of a 2D (010) crystal of brushite grown on the (010) face of the same phase (black dots on the background). Inspired by Pinto *et al.* (2009 ▸).

Fig. 2 ▸ shows a typical free-brushite morphology; however, one has to be cautious since its twinning is very common. That the brushite morphology represents a challenge is easily deduced from two important articles by Chernov *et al.* (2004 ▸, 2007 ▸), who discussed the intersection of steps coming from adjacent growth hillocks, observing them through AFM.

## 2. Twinning: the morphology of the brushite crystals

The most recurrent twin law of brushite is conveniently expressed by the twin axis [010] ≡ *A*_2_, which cannot be a symmetry element of the crystal. Hence, we have to show that the (P, T) (P, parent; T, twin) lattice misfit between parent and twinned individuals is very low, implying that its activation energy for nucleation should be very low too. Concerning the other twin laws shown by pure brushite, twinning is obtained through the planes (100) ≡ *m*_1_, (101) ≡ *m*_2_ and (001) ≡ *m*_3_. It has also been observed that, in these cases, the common cell multiplicity is low while the angular misfits are no longer negligible. Abbona *et al.* (1993 ▸) discuss the crystal-chemical aspects of the twin growth mechanisms, but they did not address the crystal complexity. We think that it is not a good strategy to make assumptions about the nature of the twinning; it would be better to search for the P and T vectors defining the 2D lattice coincidences (2D-LCs hereafter) in the common (010) twin plane. The related 2D-LC obliquity is necessary as well. All this preliminary and crystallographic evaluation is needed to define twins ‘quantitatively’ through their geometric hierarchy, along with their specific twin and adhesion energies. As an example, the reader is referred to the work of Bruno *et al.* (2010 ▸) on the four twin laws of calcite.

**Figure 3:**
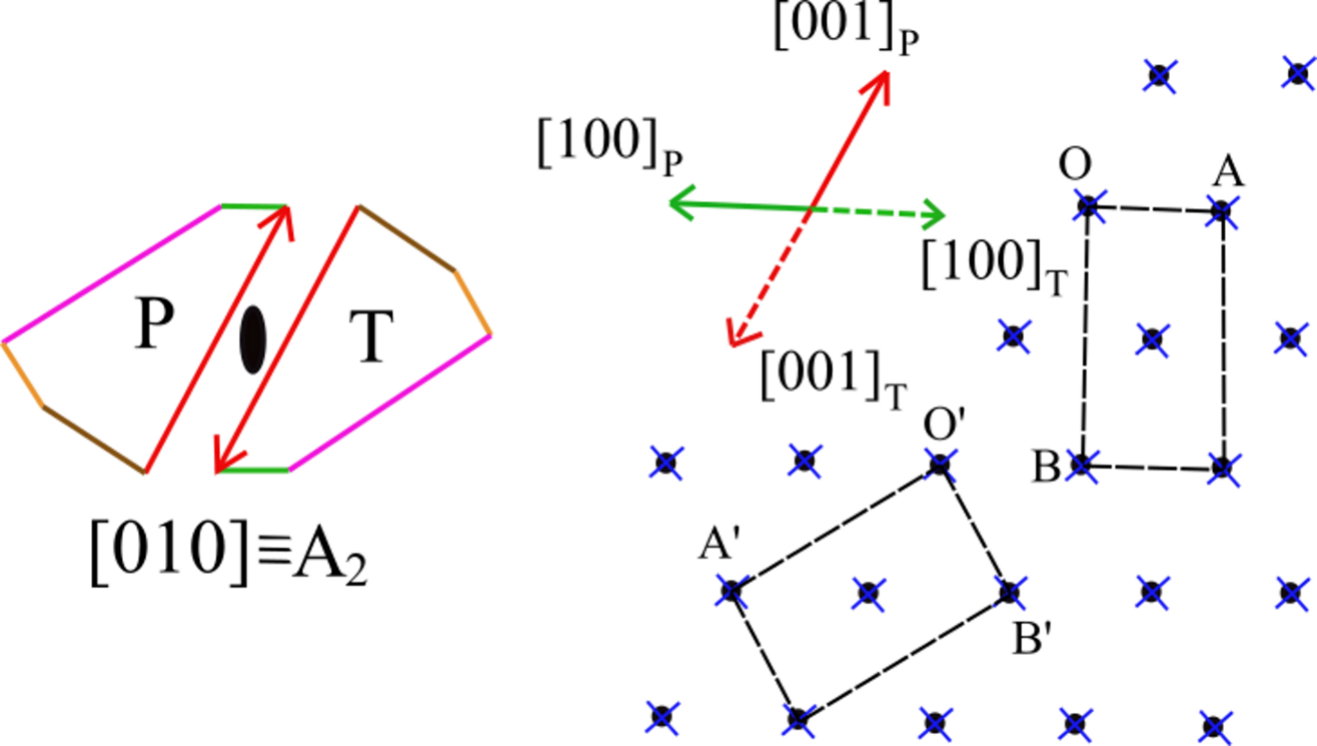
The most frequent twin law is ruled by *A*_2_ ≡ [010] ≡ twin axis, imposed by external symmetry. The brushite space group, *Aa*, does not allow the *A*_2_ ≡ [010] ≡ twin axis. Two examples of 2D-LC areas are drawn (dots and crosses refer to P and T lattices). Equivalent directions are represented with the same color.

The external symmetry, imposed by gypsum {010}, gives the law *A*_2_ ≡ [010] ≡ twin axis. From Fig. 3 ▸, one sees that P and T brushite individuals are related by [100]_P_ = −[100]_T_ and [001]_P_ = −[100]_T_. The 2D-LCs of P and T can be perfectly superposed. The common 2D-LC, expressed through the P lattice vectors, reduces to simple meshes: either OA = [100] × OB = [102], or O′A′ = [101] × O′B′= [101]. The 2D-LC occupies two meshes, corresponding to an area of 64.947 Å^2^. The obliquity always equals 0°.

**Figure 4:**
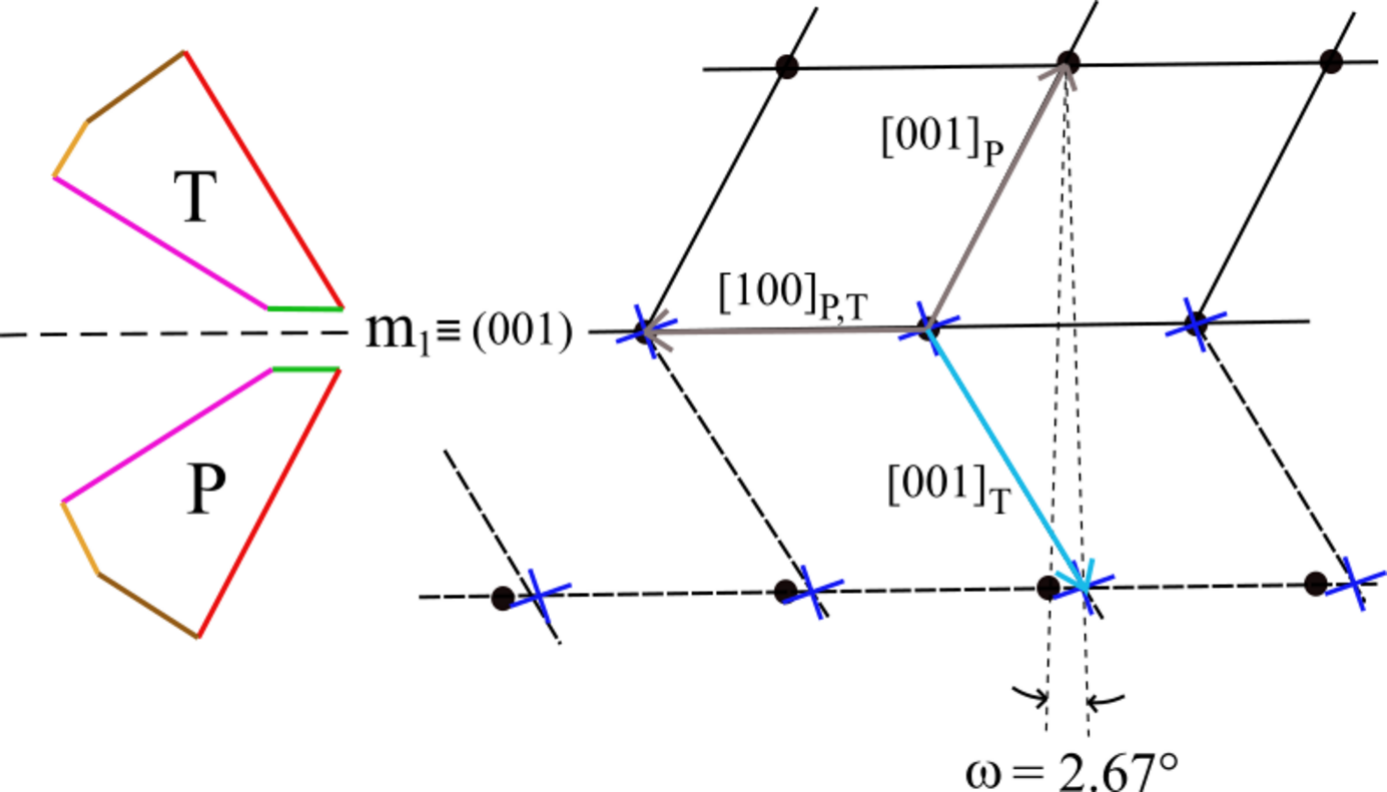
The most recurrent twin law of brushite is due to (001) ≡ *m*_1_ as a twin plane.

**Figure 5:**
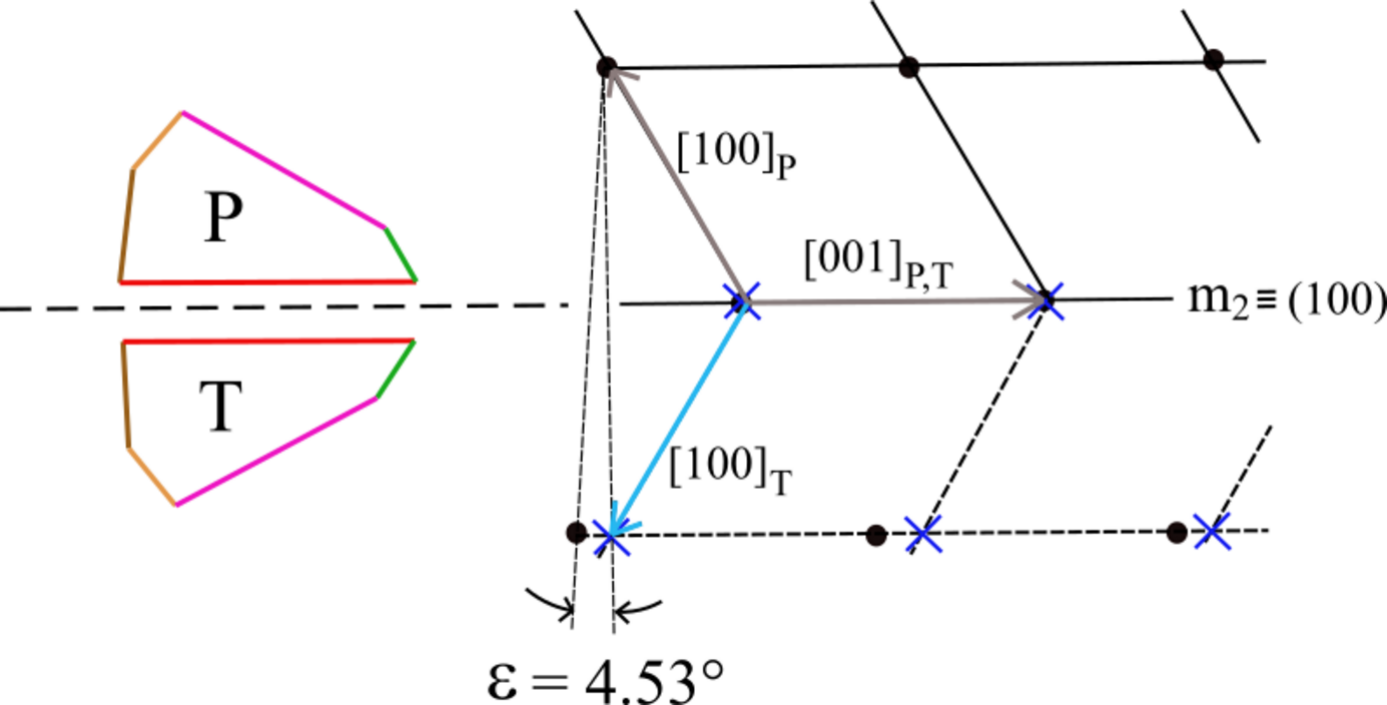
The brushite (100) = *m*_2_ twin. An angle of 118.52° is found between the vectors [001]_P,T_ and [100]_P_. The obliquity reaches a value of 4.53°.

The law (001) ≡ *m*_1_ defines a twin plane (Fig. 4 ▸). (001) ≡ *m*_1_ coincides with the [010], [100]_P,T_ mirror plane. In this case, one has two 2D-LCs. Crystal P exhibits an angle of 118.52° between the vectors [100]_P,T_ and [001]_P_. The twinned crystal T exhibits an angle of 118.52° as well, but it is between [100]_P,T_ and [001]_T_. The obliquity here reaches its smallest value of ω = 2.67°.

(100) ≡ *m*_2_ is also a twin plane (Fig. 5 ▸) which coincides with a mirror plane. The two 2D-LCs associated with P and T are defined by the P crystal, lying between the vectors [001]_P,T_ and [100]_P_; the angle between them is 118.52°. The same angle is obtained between the vectors [001]_P,T_ and [100]_T_ as well, and the obliquity between the two lattices reaches the higher value ɛ = 4.53°, which can be tolerated for an epitaxy.

**Figure 6:**
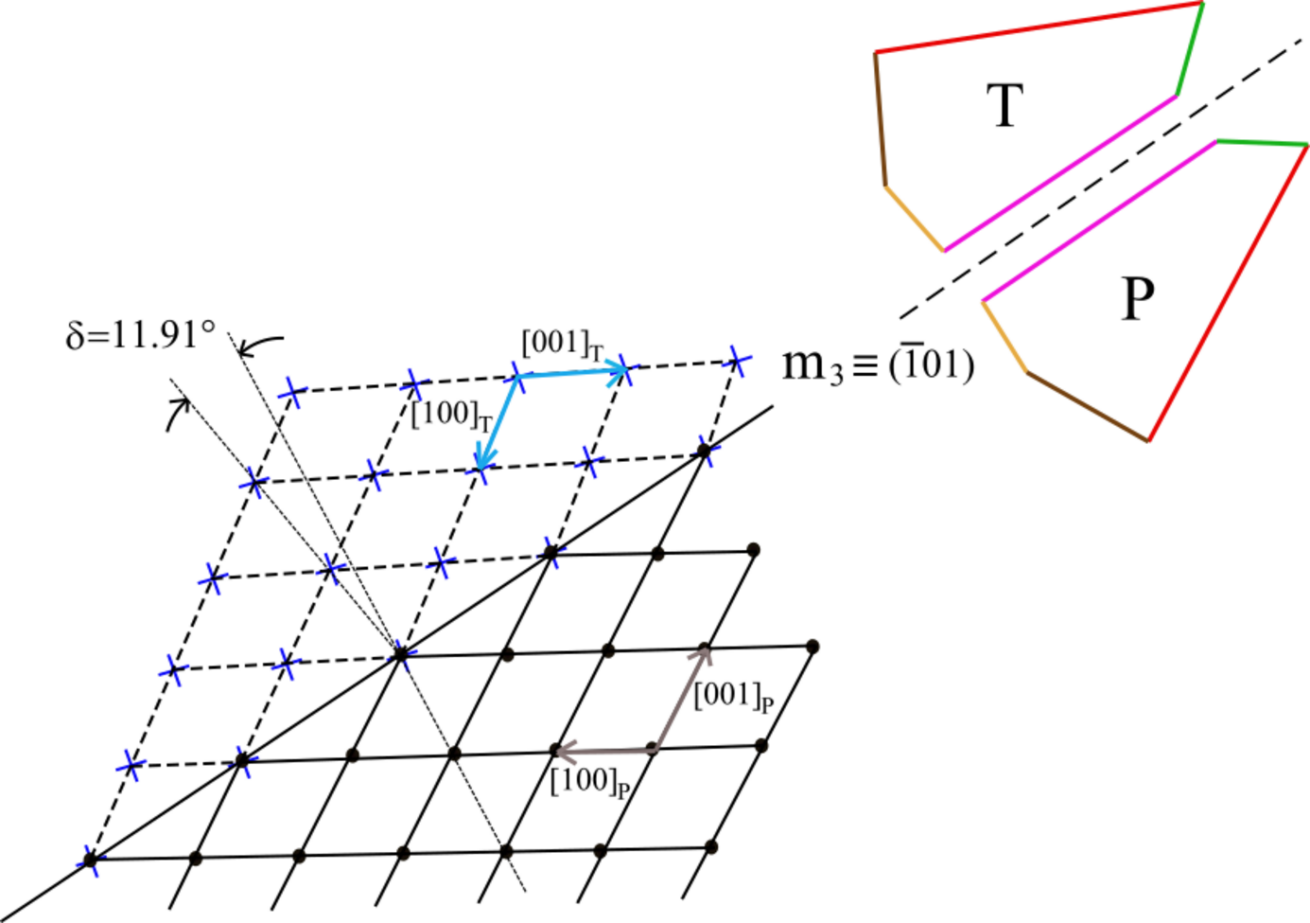
The last brushite twin. The corresponding twin law is (101) ≡ *m*_3_.

The law (101) ≡ *m*_3_ is the last among the twin planes (Fig. 6 ▸) and contains [010] and [101]_P,T_, where [101]_P,T_ is the long diagonal of the parallelogram made by the vectors [100]_P_ and [001]_P_ (with an internal angle of 118.52°). Here, the relation between these two twinned 2D-LCs becomes a little complicated: in fact, the twin plane (*m*_3_) works in such a way that the parent vector [100]_P_ is transformed into the twinned one [100]_T_ while the same twin plane changes the parent vector [001]_P_ into the corresponding one [001]_T_. If one wants to preserve the angle of 118.52° in this P–T transition, the angular misfit has to be calculated between the directions of the short cell diagonals [101]_P_ and [101]_T_. Hence, we found a large obliquity of δ = 11.91°.

When summarizing the four twins and their obliquity, one can say:

(i) The best twin is induced by the {010} form of gypsum, owing to its twofold *A*_2_ ≡ [010] axis. Its obliquity equals 0° and it is correctly called a growth twin.

(ii) The other three are normal twins, generated by twin planes, and their occurrence probability will follow the related obliquities: (001) ≡ *m*_1_ → obliquity = 2.67°; (100) ≡ *m*_2_ → obliquity = 4.53°; (101) ≡ *m*_3_ → obliquity = 11.91°.

These calculations do not agree with the past conclusions (Abbona *et al.*, 1993 ▸) about the gypsum and brushite twin laws on their common (010) plane. Having considered the strong similarities between their lattices, we can affirm that gypsum and brushite twins can be easily distinguished (Rubbo *et al.*, 2012*a* ▸; Rubbo *et al.*, 2012*b* ▸), since the gypsum obliquities (G_obl_) are ranked in another way: swallowtail (100) ≡ *m*_1_ → 0.6 < G_obl_ < 2.3; Montmartre (101) ≡ *m*_2_ → 0.09 < G_obl_ < 2.19; (001) ≡ *m*_3_ → G_obl_ = 2.92; (201) ≡ *m*_4_ → G_obl_ = 2.92; (101) ≡ *m*_5_ → G_obl_ = 12.80.

**Figure 7:**
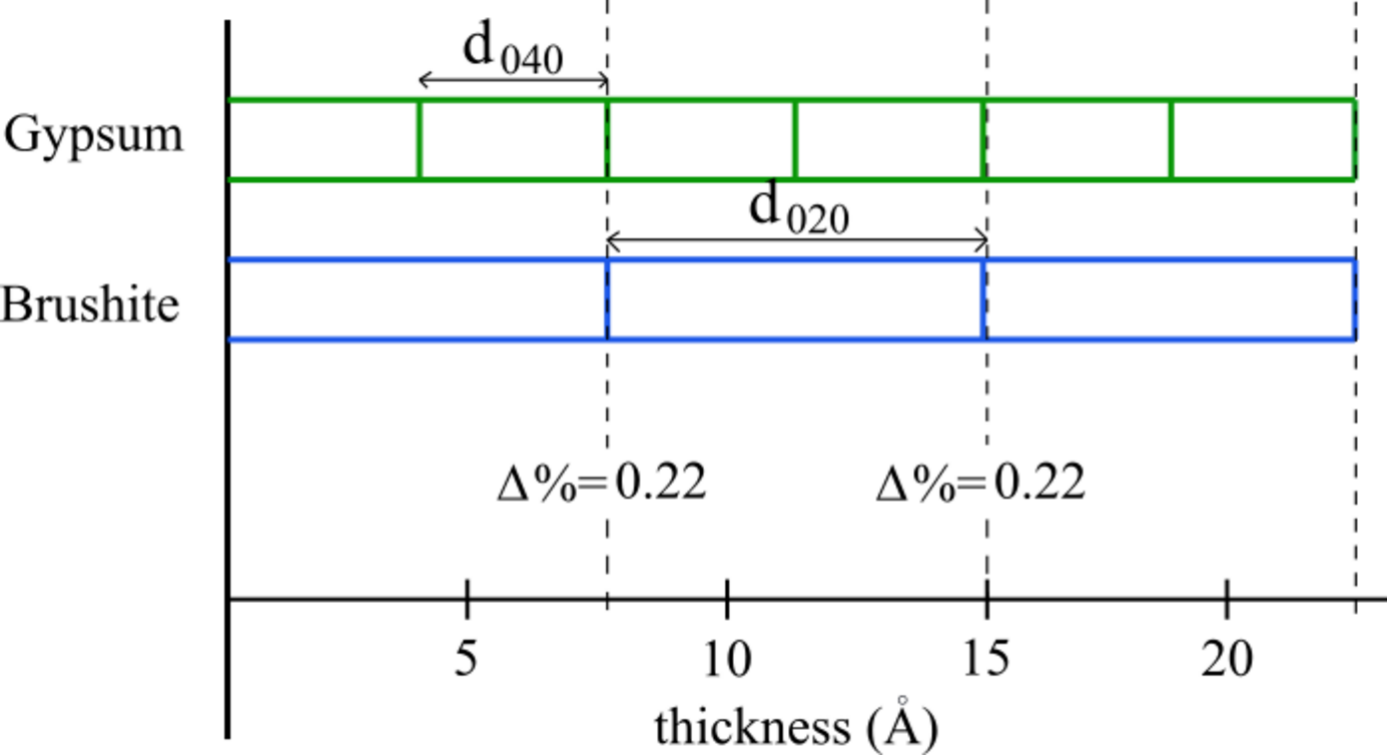
A comparison of the *d*_0*k*0_ gypsum and brushite growth layers (Aquilano *et al.*, 2022 ▸; see Table 1 ▸).

Gypsum {010} generates brushite {010}. A comparison between the thicknesses of elementary growth layers of gypsum and brushite on the common 010 plane (layer thickness in Å) is shown in Fig. 7 ▸ and Table 1 ▸. The related outcropping Burgers vectors are the same.

The lowest gypsum layer thickness *d*_040_ ∼ 3.803 Å was first observed and measured (AFM) by Aquilano *et al.* (2022 ▸). According to the opinion expressed by Pinto *et al.* (2009 ▸): ‘…the fact that brushite forms thin platelets instead of thick 3D-crystals [see, *e.g.*: pharmacolite by Rodríguez-Blanco *et al.* (2007 ▸)] can be explained by the better brushite/gypsum fit. The observation points towards a Stranski–Krastanow mechanism rather than a Volmer–Weber epi-growth and suggests a 2D-reticular control, with [001] and [100] as the main matching directions.’

In our opinion, and referring to both Table 1 ▸ for the *d*_0l0_ thicknesses and the clear picture reported by Pinto *et al.* (2009 ▸), [101] brushite coincides with the [101] direction of gypsum. This agrees with our 2D-LC rank 1a (Table 2 ▸) at the (010) interface between gypsum and brushite. Pinto *et al.* (2009 ▸) showed that the strong bond network within the *d*_020_ brushite layers points towards the dominating {010} flat pinacoid, which agrees with their observed morphologies. Then, in the early stages of growth, these crystals are so thin that their lateral forms are difficult to distinguish, their contours being analogous to those of the irregular crystals obtained by Abbona *et al.* (1993 ▸) from the precipitation of pure brushite. With increasing growth time, the {010} brushite platelets, even if they remain thin, develop well defined contours, allowing an angle of 95.98° between the forms {111} and {111}. These directions are [101] brushite and [101] gypsum, respectively. This morphology corresponds to that of the precipitation obtained in the presence of SO_4_^2−^ ions at relatively high supersaturation by Rinaudo *et al.* (1994 ▸). The forms {111}, {111} and {120}, along with other minor forms, are reported by Abbona *et al.* (1993 ▸), as also indicated by Pinto *et al.* (2009 ▸).

**Figure 8:**
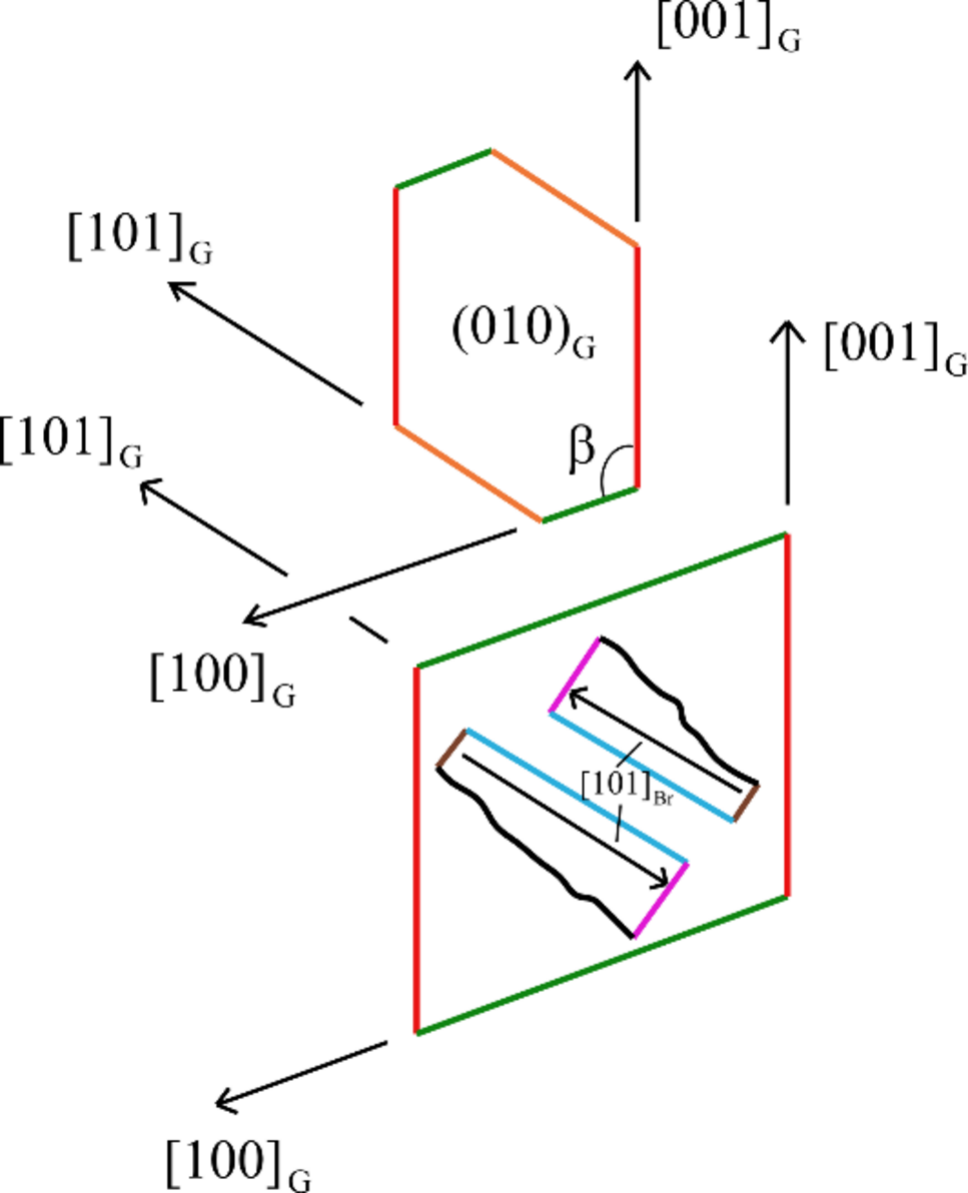
(Upper) The {010} form of gypsum (G) where the [100], [001] and [101] directions are represented; the angle is 114.08°. (Lower) The same gypsum {010} form, but brushite platelets (Br) have been superposed: direction [101]_G_ is parallel to [101]_Br_. Owing to the brushite space group (*Aa*), [101]_Br_ is not symmetry equivalent to the [101]_Br_ direction.

In the present work, we are dealing with the kind of solvent-mediated transformation observed by Cardew & Davey (1985 ▸); this replacement of gypsum by brushite was viewed by Pinto *et al.* (2009 ▸) as a true epitaxy, according to the correct classification by Bonev (1972 ▸), because the guest brushite grows on the surface of dissolving host gypsum (see Fig. 8 ▸, concerning the reciprocal orientations of guest–host crystals). Here, in gypsum, which is (*A*2/*a*) center-symmetric, the positive direction [101] is identical to the negative [101] one. But this is no longer true for brushite, which belongs to the polar space group *Aa*, where [101] and its opposite [101] are not equivalent. In an elegant experiment, Pinto *et al.* (2009 ▸) showed that the brushite platelets grow epitaxially on gypsum, linked by the *A*_2_ axis, but they are mutually polar and not center-symmetric. Hence, this aggregate of platelets builds an observable ‘twin-related’ crystal set, the law being [010] ≡ twin axis or (001) ≡ twin plane.

## 3. Several contradictions in the structural fit of gypsum–brushite on their (010) plane

Concerning the structural match between gypsum and brushite, it is worth pointing out the differences between the approach of Rodríguez-Blanco *et al.* (2007 ▸) and Pinto *et al.* (2012 ▸) and our way of conceiving the epitaxy. For Pinto *et al.* (2012 ▸), it is fundamental to consider the per cent misfit, mf(%), between pairs of the envisaged structures:
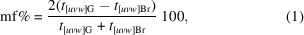
where 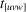 is the repeating period along the [*uvw*] direction. On this basis, Pinto *et al.* (2009 ▸) affirmed that ‘…all these misfits are clearly within the limits required for the epi-nucleation from solution, as in Walton (1969 ▸), which justifies the development of an oriented overgrowth of brushite on the gypsum cleavage surface’.

In the present study, we adopted a different strategy to evaluate all the existing 2D-CL per cent misfits, as will be described below.

At this point, confusion can arise between the vector’s misfits and the differences in the thicknesses of elementary growth layers of gypsum and brushite on the (010) plane. As previously discussed, layer thicknesses and vector misfits can be treated separately since they are independent of each other.

Suppose we obtain a 2D-LC between two superposed lattices (1 and 2), making a (tentative) epitaxy: the 2D-LC vectors being **t**_1a_, **t**_1b_ and **t**_2a_, **t**_2b_. Thus, we can define the linear misfits
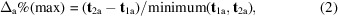

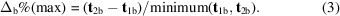


Accordingly, the 2D area (Å^2^) is calculated for both meshes (**t**_1a_, **t**_1b_) and (**t**_2a_, **t**_2b_). Then, one can obtain the maximum per cent area misfit [equation (4)] between the two meshes:
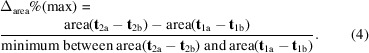


Moreover, a crucial parameter is the angular misfit (obliquity) between the superposed 2D-LCs. The related obliquity should be small if the linear misfits (2) and (3) have opposite signs. Otherwise, when the linear misfits have coherent signs, the obliquity value risks rising over 5°; accordingly, the 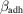 value related to the hypothetical epitaxy indicates that this epitaxy is hindered.

Summing up, our constraints on the epitaxy (linear or area misfits ≤ 5%, obliquity ≤ 5°) are rather strict, in relation not only to Pinto’s results but to previous ones as well. This was due neither to a geometric nor to a reticular preconception but only to our recent observations. In particular, we considered (*a posteriori*) the strategic importance of a physical parameter such as 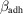, ruling in a complex way all these epi-phenomena, as has been shown experimentally (Aquilano & Pastero, 2013 ▸; Aquilano *et al.*, 2022 ▸; Aquilano *et al.*, 2023 ▸).

Coming back to Table 2 ▸, the modifications introduced by our rank 1a are not dramatic, as concerns the directions [001] and [100] of the basic cell. Values of mf(%) = −1.265 for [001] and −2.332% for [100] have been obtained by Pinto’s method. Instead, we obtained mf(%) = −1.16% and mf(%) = −2.36%, respectively. As for the angular misfit, we think that the value of 3.8° obtained by Pinto *et al.* (2009 ▸) is not valid, since the angular misfit is due to the facile angular difference (β′ − β), and then only our value (4.44°) is correct. As regards the difference between the 2D areas, we obtained a very reasonable value (+0.35%), for which there are no references in the other method. In conclusion, Pinto’s reasoning and our 2D-LC method (rank 1a) practically agree; nevertheless, the angular misfit that we calculated does not permit an easy 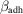 calculation of the pair (010) gypsum/(010) brushite, owing to the unavoidable situation that the linear vector misfits have the same sign (full coherence means lattice divergence) and that the just mentioned angular misfit (4.44°) adds to a borderline epitaxy.

In contrast, an unexpected situation of epitaxy occurs when one considers alternative 2D-LCs, as presented in Table 2 ▸ (rank 2). The rotation angle between (010) gypsum and (010) brushite remains 0°, but in this case [101] gypsum // [101] brushite and [101] gypsum // [101] brushite. Here, the areas of the 2D-LC mesh are twice the preceding ones, but the linear misfits have opposite signs (vector incoherence means lattice convergence), and the obliquity reaches a negligible value (<0.4°). This proves again that only the coexistence of a few rigorous constraints, applied to the common 2D-LCs, allows us to perform credible evaluations of 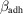, the unique physical parameter to be considered when dealing with 2D-LCs.

## 4. Conclusion

Following the mixed (epitaxy–twinning) mechanism, the {010} epitaxy observed between non-polar gypsum and polar brushite crystals has been reviewed.

First, an order has been found in the brushite twinning of the five {010} gypsum/brushite interfaces. Hence, the obliquity criterion applied to the 2D-LCs resulted in it being necessary to distinguish gypsum from brushite twins.

Secondly, and for the first time in brushite/gypsum growth, we observed:

(*a*) The vectors normal to the (010) layers in gypsum and brushite are rigorously parallel.

(*b*) The extraordinary coincidence (Δ% < 0.3) of the *d*_020_ thickness of the superposing layers of brushite growing onto the transforming gypsum is further proof that this epitaxy points towards a complex mechanism of adsorption/absorption, as proposed by Aquilano & Pastero (2013 ▸). To gain a deeper understanding of and differentiate between these epi-mechanisms, it will be fundamental to examine the texts of Mutaftschiev (2013 ▸, 2002 ▸, 1981 ▸) devoted to crystal growth.

Lastly, we outlined the differences between Pinto’s rule (as an example) and our way of conceiving the epitaxy. We are more restrictive than the previous approaches on (i) linear misfits and coherence of the 2D-LC parameters; (ii) differences in the common areas of the related 2D-LCs; (iii) their obliquities. These new simple criteria allowed us to distinguish between a true or faulted epitaxy and carry out an evaluation of 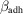, the sole meaningful epi-parameter.

## Figures and Tables

**Table 1:** The observed thickness of the elementary {010} gypsum layers and the {010} brushite layers Their very low per cent difference (Δ%) is striking.

Gypsum	Thickness (Å)	Brushite	Thickness (Å)	Δ%
*d*_040_	3.803			
2*d*_040_	7.606	*d*_020_	7.590	−0.22
3*d*_040_	11.409			
*d*_010_	15.213	*d*_010_	15.180	−0.22

**Table 2:** 2D-LCs between the monoclinic {010} gypsum and {010} brushite The 2D-LCs are sorted (‘Rank’) according to their increasing area. Maximum linear and 2D-LC area misfits are presented with their obliquities (angular misfit). 2D cell parameters (Å) and 2D cell area (Å^2^) are also provided. Gypsum: *a*_0_ = 5.678, *b*_0_ = 15.213, *c*_0_ = 6.286 Å, β = 114.08° (space group *A*2/*a*); brushite: *a*_0_ = 5.812, *b*_0_ = 15.180, *c*_0_ = 6.359; β′ = 118.52° (space group *Aa*).

	Rank	(010) Brushite	(010) Gypsum	Maximum misfit Δ%	Angular misfit (°)	Rotation angle (°)
2D-LC parameters	1a	[001] = 6.359	[001] = 6.286	−1.16		
		[100] = 5.812	[100] = 5.678	−2.36		
2D-LC area		32.47 (1×)	32.59 (1×)	+0.35	4.44	0°

2D-LC parameters	1b	[001] = 6.359	[100] = 5.678	−11.99		
		[100] = 5.812	[001] = 6.286	+2.36		
2D-LC area		32.47 (1×)	32.59 (1×)	+0.35	4.44	

2D-LC parameters	2	[101] = 6.240	[101] = 6.520	+4.49		
		[101] = 10.465	[101] = 10.044	−4.19		
2D-LC area		64.9472 (2×)	65.17175 (2×)	+0.35	0.40	0°

2D-LC parameters	3	[101] = 6.24	[001] = 6.286	+0.74		
		[102] = 16.313	−3 × [100] = 17.034	+4.42		
2D-LC area		113.96 (3×)	97.76 (3×)	−16.57	7.28	

2D-LC parameters	4a	[101] = 6.24	[001] = 6.286	+0.74		
		[301] = 21.221	2 × [201] = 20.997	−1.07		
2D-LC area		129.89 (4×)	130.34 (4×)	+0.35	2.12	52°

2D-LC parameters	4b	[102] = 11.1777	2 × [100] = 11.356	−1.60		
		2 × [100] = 11.624	[102] = 11.491	+1.16		
2D-LC area		129.89 (4×)	130.34 (4×)	+0.35	1.40	90°

## References

[bb1] Abbona, F., Calleri, M., Franchini-Angela, M. & Ivaldi, G. (1994). *N. Jahrb. Miner. Abh.* **168**, 171–184.

[bb2] Abbona, F., Christensson, F., Angela, M. F. & Madsen, H. E. L. (1993). *J. Cryst. Growth* **131**, 331–346.

[bb3] Aquilano, D., Bruno, M., Cotellucci, A., Pastero, L. & Ghignone, S. (2022). *CrystEngComm* **24**, 5120–5127. 10.1107/S1600576722008196 PMC9533751 36249497

[bb4] Aquilano, D., Bruno, M., Ghignone, S., Pastero, L. & Cotellucci, A. (2023). *J. Appl. Cryst.* **56**, 338–348. 10.1107/S1600576722008196 PMC9533751 36249497

[bb5] Aquilano, D., Otálora, F., Pastero, L. & García-Ruiz, J. M. (2016). *Prog. Cryst. Growth Charact. Mater.* **62**, 227–251.

[bb6] Aquilano, D. & Pastero, L. (2013). *Cryst. Res. Technol.* **48**, 819–839.

[bb7] Bonev, I. (1972). *Acta Cryst.* A**28**, 508–512.

[bb8] Bosbach, D. & Hochella, M. F. Jr (1996). *Chem. Geol.* **132**, 227–236.

[bb9] Bosbach, D. & Rammensee, W. (1994). *Geochim. Cosmochim. Acta* **58**, 843–849.

[bb10] Bruno, M., Massaro, F. R., Rubbo, M., Prencipe, M. & Aquilano, D. (2010). *Cryst. Growth Des.* **10**, 3102–3109.

[bb11] Cardew, P. T. & Davey, R. J. (1985). *Proc. R. Soc. London A* **398**, 415–428.

[bb12] Chernov, A. A., De Yoreo, J. J. & Rashkovich, L. N. (2007). *J. Optoelectronics Adv. Mater.* **9**, 1191–1197.

[bb13] Chernov, A. A., De Yoreo, J. J., Rashkovich, L. N. & Vekilov, P. G. (2004). *MRS Bull.* **29**, 927–934.

[bb14] Criado-Reyes, J., Pastero, L., Bruno, M., García-Ruiz, J. M., Aquilano, D. & Otálora, F. (2020). *Cryst. Growth Des.* **20**, 1526–1530.

[bb15] De Yoreo, J. J. (2003). *Rev. Mineral. Geochem.* **54**, 57–93.

[bb16] Heijnen, W. M. M. & Hartman, P. (1991). *J. Cryst. Growth* **108**, 290–300.

[bb18] Le Geros, R. Z. & Le Geros, J. P. (1972). *J. Cryst. Growth* **13/14**, 476–480.

[bb19] Lundager Madsen, H. E. (2008). *J. Cryst. Growth* **310**, 2602–2612.

[bb20] Lundager Madsen, H. E. & Thorvardarson, G. (1984). *J. Cryst. Growth* **66**, 369–376.

[bb21] Ma, G. & Liu, X. Y. (2009). *Cryst. Growth Des.* **9**, 2991–2994.

[bb22] Massaro, F. R., Rubbo, M. & Aquilano, D. (2010). *Cryst. Growth Des.* **10**, 2870–2878.

[bb23] Monier, J. C. & Kern, R. (1956). *Bull. Soc. Fr. Minéral. Cristallogr.* **79**, 495–514.

[bb26] Mutaftschiev, B. (1981). *Interfacial aspects of phase transformations*. NATO Advanced Study Institutes. Erice, Sicily, Italy.

[bb25] Mutaftschiev, B. (2002). *Crystal growth: from large to small*, in *Joint Italo-French meeting, Rome*, pp. 13–30. Accademia Nazionale dei Lincei.

[bb24] Mutaftschiev, B. (2013). *Cryst. Res. Technol.* **48**, 706–726.

[bb27] Pastero, L. & Aquilano, D. (2016). *Cryst. Growth Des.* **16**, 852–860.

[bb28] Pinto, A. J., Carneiro, J., Katsikopoulos, D., Jiménez, A. & Prieto, M. (2012). *Cryst. Growth Des.* **12**, 445–455.

[bb29] Pinto, A. J., Jimenez, A. & Prieto, M. (2009). *Am. Mineral.* **94**, 313–322.

[bb30] Pinto, A. J., Ruiz-Agudo, E., Putnis, C. V., Putnis, A., Jimenez, A. & Prieto, M. (2010). *Am. Mineral.* **95**, 1747–1757.

[bb31] Rinaudo, C., Lanfranco, A. M. & Boistelle, R. (1996). *J. Cryst. Growth* **158**, 316–321.

[bb32] Rinaudo, C., Lanfranco, A. M. & Franchini-Angela, M. (1994). *J. Cryst. Growth* **142**, 184–192.

[bb33] Rodríguez-Blanco, J. D., Jiménez, A. & Prieto, M. (2007). *Cryst. Growth Des.* **7**, 2756–2763.

[bb34] Rubbo, M., Bruno, M., Massaro, F. R. & Aquilano, D. (2012*a*). *Cryst. Growth Des.* **12**, 264–270.

[bb35] Rubbo, M., Bruno, M., Massaro, F. R. & Aquilano, D. (2012*b*). *Cryst. Growth Des.* **12**, 3018–3024.

[bb36] Van Driessche, A. E. S., García-Ruiz, J. M., Delgado-López, J. M. & Sazaki, G. (2010). *Cryst. Growth Des.* **10**, 3909–3916.

[bb37] Walton, A. G. (1969). *Nucleation*, edited by A. C. Zettlemoyer, pp. 225–307. New York: Marcel Dekker.

